# Supplementary material for: Rhesus monkeys use both eye and head gaze to reallocate covert spatial attention facilitating visual perception
Source: Cogn Affect Behav Neurosci. 2026 Mar 25;26(3):1036–53. doi: 10.3758/s13415-025-01383-0 (PMC13260114; doi:10.3758/s13415-025-01383-0)

**Rhesus monkeys use both eye and head gaze to reallocate covert spatial attention facilitating visual perception**

**Figure S1**

*(A) Schematic of the task used for perceptual threshold assessment. (B) Psychometric functions describing the association between the hit rate and luminance change detection for monkey E and C. Stimulus level refers to a total of 512 steps in which the luminance of the LEDs could be varied. The number of trials was 1820 for monkey E and 2218 for monkey C.*


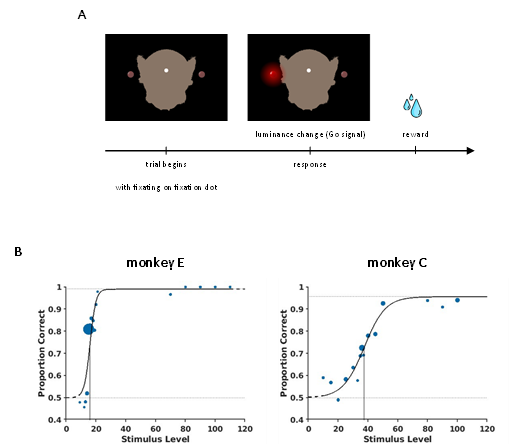


**Table S1.**

*GLMM and ANOVA outputs for the fixed predictors in the eye-only gaze condition.*

|  | monkey E ^a^ | |  | monkey C ^b^ | |
| --- | --- | --- | --- | --- | --- |
|  | ${Wald \chi}^{2}$ | $log$-$odds$ |  | ${Wald \chi}^{2}$ | $log$-$odds$ |
| intercept | 337.15 *** | 1.95 *** |  | 170.17 *** | -0.92*** |
| congruency (ref: incongreunt) | 5.13 * | -0.34 *** |  | 0.27 | -0.04 |
| SOA (ref: 50) | 66.33 *** | - |  | 0.31 | - |
| 100 | - | -0.50 *** |  | - | - |
| 200 | - | -0.78 *** |  | - | 0.01 |
| 300 | - | -0.61 *** |  | - | - |
| 400 | - | -0.85 *** |  | - | -0.05 |
| SOA x congruency | 7.07 | - |  | 2.00 | - |
| 100:congruency | - | -0.08 |  | - | - |
| 200:congruency | - | -0.12 |  | - | -0.13 |
| 300:congruency | - | -0.24 * |  | - | - |
| 400:congruency | - | -0.19 |  | - | -0.05 |
| SOA x target direction | 291.26 *** | - |  | 179.84 *** | - |
| 100:target direction | - | -0.20 *** |  | - | - |
| 200:target direction | - | -0.56 *** |  | - | -0.64*** |
| 300:target direction | - | -0.46 *** |  | - | - |
| 400:target direction | - | -0.47 *** |  | - | -0.83*** |
| SOA x normalized log RT | 795.14 *** | - |  | 264.95 *** | - |
| 100: normalized log RT | - | -0.32 *** |  | - | - |
| 200: normalized log RT | - | -0.40 *** |  | - | -0.41*** |
| 300: normalized log RT | - | -0.40 *** |  | - | - |
| 400: normalized log RT | - | -0.35 *** |  | - | -0.39*** |
| ^a^ number of observations: 33751; random predictor (session number = 63): variance = 0.047; adjusted ICC = 0.014  ^b^ number of observations: 10202; random predictor (session number = 13): variance = 0.012; adjusted ICC = 0.004  * p<0.05 ** p<0.01 *** p<0.001 | | | | | |

**Figure S2**

***Experimental paradigm illustrating trial structure, observer behavior, and associated neural mechanisms for covert and overt attentional shifts.*** *(a) At trial onset, the observer is required to maintain fixation (green dashed lines) on the central fixation point (CF) while viewing an avatar with direct gaze. During the gaze-cue epoch, the avatar shifts its gaze toward one of the two peripheral LEDs, while the observing monkey must continue fixating on the CF, preventing any overt attentional shift. Subsequently, a brief luminance change at one of the LEDs prompts the observer to execute a saccade (green dashed lines) toward the target LED. (b–c) Throughout the direct-gaze and gaze-shift events, the observer is required to maintain strict central fixation; violations result in immediate trial abortion with no reward. Only after the luminance change is the observer permitted to break fixation and make a saccade to the target LED. (d) During the gaze-cue epoch, the avatar’s directed gaze can induce a covert attentional shift in the observer. Although the observer’s eyes remain fixed on the CF, attention is internally allocated toward the gaze-cued location (depicted as the “spotlight of attention” in panel (a)). This covert shift reflects a selective enhancement of the retinal signals arising from the peripheral regions of the retina corresponding to the cued location (the fovea must remain aligned with the CF). When the subsequent luminance change appears at the gaze-congruent LED, its image falls on the same region of the retinal periphery that has already been prioritized, thereby facilitating processing of the perceptually challenging luminance change and improving detection accuracy. In contrast, during gaze-incongruent trials (example in panel (a)), the attentional spotlight is misallocated toward the distractor LED, reducing sensitivity to the luminance change presented on the opposite side and consequently lowering detection accuracy.*


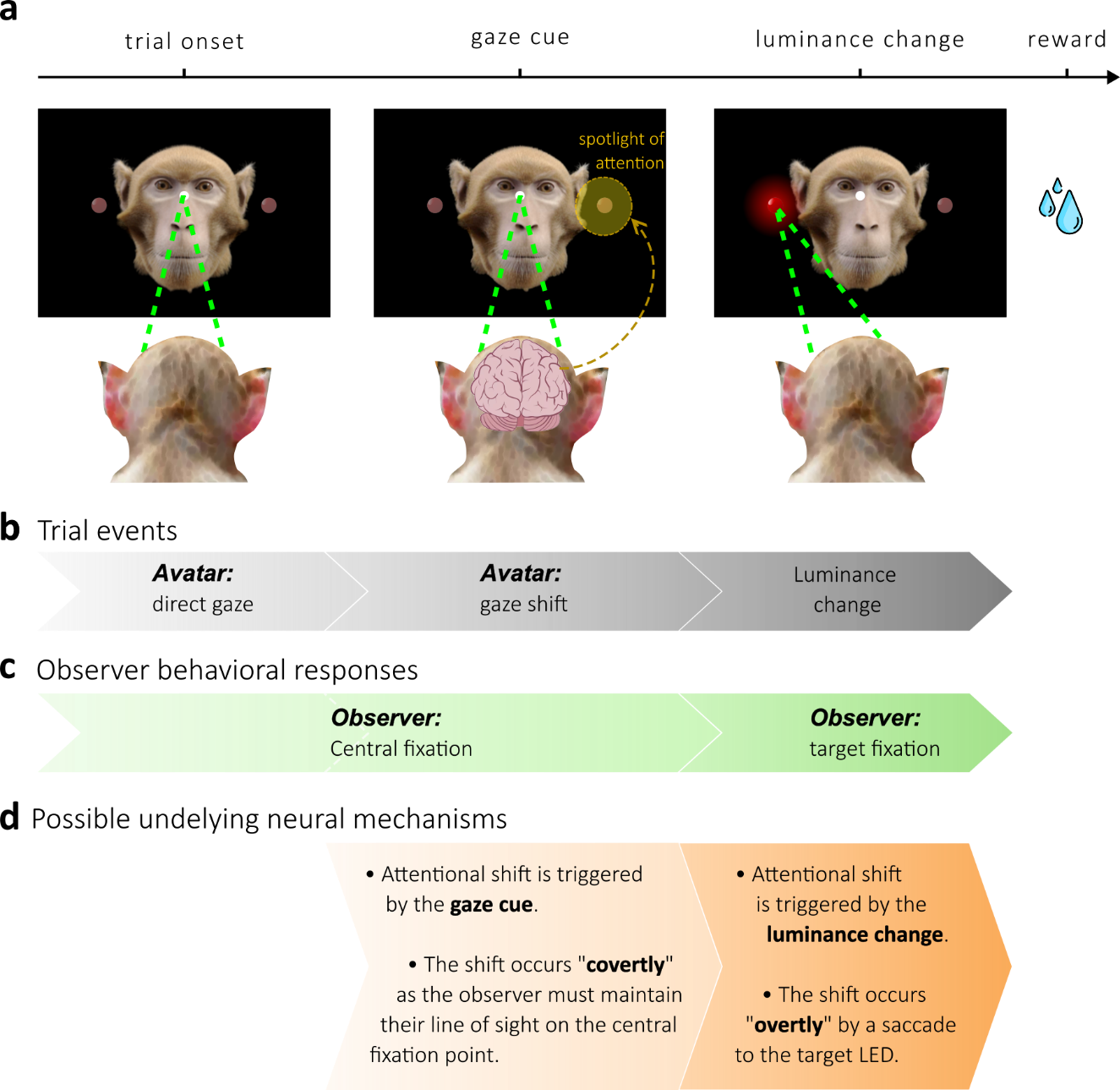

Supplement: Supplementary file 1 — Supplementary file1 (DOCX 679 kb) [file 13415_2025_1383_MOESM1_ESM.docx]
